# Retracted: Study on the Mechanism of Platelet-Released Clusterins Inducing Restenosis after Carotid Endarterectomy by Activating TLR3/NF-*κ*b p65 Signaling Pathway

**DOI:** 10.1155/2023/9784573

**Published:** 2023-02-02

**Authors:** Journal of Healthcare Engineering

*Journal of Healthcare Engineering* has retracted the article titled “Study on the Mechanism of Platelet-Released Clusterins Inducing Restenosis after Carotid Endarterectomy by Activating TLR3/NF-*κ*b p65 Signaling Pathway” [1] due to concerns that the peer review process has been compromised.

Following an investigation conducted by the Hindawi Research Integrity team [2], significant concerns were identified with the peer reviewers assigned to this article; the investigation has concluded that the peer review process was compromised. We therefore can no longer trust the peer review process, and the article is being retracted with the agreement of the Chief Editor.

The authors do not agree to the retraction.
